# Response to correspondence from Koerner and colleagues concerning our paper entitled: The effect of diluting povidone-iodine on bacterial growth associated with speech

**DOI:** 10.1186/s12886-020-01599-3

**Published:** 2020-08-12

**Authors:** Sivashanth Gnanasekaran, Sophie Rogers, Sanj Wickremasinghe, Sukhpal S. Sandhu

**Affiliations:** Centre For Eye Research Australia, The Royal Victorian Eye and Ear Hospital, University of Melbourne, 32 Gisborne Street, East Melbourne, Victoria 3002 Australia

**Keywords:** Anti-VEGF, Endophthalmitis, Intravitreal injection, Povidone-iodine, Pre-injection antisepsis

## Abstract

Clinicians adopt varying strategies for antisepsis with PI, which to this day remains efficient, economical and effective. Clinicians should prudently consider effective PI application, and we thank Koerner and Grzybowski for encouraging debate and raising the profile of this issue.

## Main text

We thank Koerner and Grzybowski [1] for their comments regarding the study on the effect of diluting povidone-iodine (PI) on bacterial growth associated with speech [2]. We agree that there are no standardized reliable ways to simulate the ocular surface to analyse this in vitro, and stress that the emphasis of this study design is to add to the evidence base. This study demonstrated the differences in PI dilution and significant bacterial culture growth with bacterial droplet dispersal associated with speech. Whilst there is evidence to suggest lower doses of PI is effective [3], we note that other evidence suggest that these lower doses require several applications [4]. Clinicians adopt varying strategies for antisepsis with PI, which to this day remains efficient, economical and effective [5]. Clinicians should prudently consider effective PI application, and we again thank Koerner and Grzybowski for encouraging debate and raising the profile of this issue.

## Data Availability

The dataset used and/or analysed during the current study are available from the corresponding author on reasonable request. All data generated or analysed during this study are included in this published article.

## Abbreviation

PI: poviodone-iodine
